# Clustering Insomnia Patterns by Data From Wearable Devices: Algorithm Development and Validation Study

**DOI:** 10.2196/14473

**Published:** 2019-12-05

**Authors:** Sungkyu Park, Sang Won Lee, Sungwon Han, Meeyoung Cha

**Affiliations:** 1 Graduate School of Culture Technology Korea Advanced Institute of Science and Technology Daejeon Republic of Korea; 2 Kyungpook National University Chilgok Hospital Daegu Republic of Korea; 3 School of Computing Korea Advanced Institute of Science and Technology Daejeon Republic of Korea; 4 Data Science Group Center for Mathematical and Computational Sciences Institute for Basic Science Daejeon Republic of Korea

**Keywords:** insomnia, precision psychiatry, cluster analysis, time-series data, unsupervised learning, convolutional autoencoder

## Abstract

**Background:**

As societies become more complex, larger populations suffer from insomnia. In 2014, the US Centers for Disease Control and Prevention declared that sleep disorders should be dealt with as a public health epidemic. However, it is hard to provide adequate treatment for each insomnia sufferer, since various behavioral characteristics influence symptoms of insomnia collectively.

**Objective:**

We aim to develop a neural-net based unsupervised user clustering method towards insomnia sufferers in order to clarify the unique traits for each derived groups. Unlike the current diagnosis of insomnia that requires qualitative analysis from interview results, the classification of individuals with insomnia by using various information modalities from smart bands and neural-nets can provide better insight into insomnia treatments.

**Methods:**

This study, as part of the precision psychiatry initiative, is based on a smart band experiment conducted over 6 weeks on individuals with insomnia. During the experiment period, a total of 42 participants (19 male; average age 22.00 [SD 2.79]) from a large university wore smart bands 24/7, and 3 modalities were collected and examined: sleep patterns, daily activities, and personal demographics. We considered the consecutive daily information as a form of images, learned the latent variables of the images via a convolutional autoencoder (CAE), and clustered and labeled the input images based on the derived features. We then converted consecutive daily information into a sequence of the labels for each subject and finally clustered the people with insomnia based on their predominant labels.

**Results:**

Our method identified 5 new insomnia-activity clusters of participants that conventional methods have not recognized, and significant differences in sleep and behavioral characteristics were shown among groups (analysis of variance on rank: F_4,37_=2.36, *P*=.07 for the sleep_min feature; F_4,37_=9.05, *P*<.001 for sleep_efficiency; F_4,37_=8.16, *P*<.001 for active_calorie; F_4,37_=6.53, *P*<.001 for walks; and F_4,37_=3.51, *P*=.02 for stairs). Analyzing the consecutive data through a CAE and clustering could reveal intricate connections between insomnia and various everyday activity markers.

**Conclusions:**

Our research suggests that unsupervised learning allows health practitioners to devise precise and tailored interventions at the level of data-guided user clusters (ie, precision psychiatry), which could be a novel solution to treating insomnia and other mental disorders.

## Introduction

Approximately 30% of contemporary people have one or more symptoms of insomnia, and insomnia sufferers encounter difficulty falling or staying asleep [1,2]. Work schedule; sleep irregularity; naps; and nicotine, alcohol, and caffeine consumption can have significant effects on insomnia symptoms [3,4]. While an individual’s intermixed behavioral characteristics might affect sleep behaviors, these are not considered in current diagnostic systems such as the *Diagnostic and Statistical Manual of Mental Disorders, Fifth Edition* or the International Classification of Diseases.

With concepts of precision psychiatry emerging, individual characteristics including genetic or neuroimaging, behavioral characteristics, and individual symptoms of illness are being used to make better decisions for diagnosis or treatment. Advances in machine learning and deep learning techniques can make precision psychiatry possible in clinical situations. For instance, unsupervised learning has been applied to distinguish traits of patients with various psychiatric disorders from those of healthy subjects [5,6]. When considering the heterogeneous characteristics of insomnia patients, an approach using precision psychiatry concepts can help develop better treatment methods for insomnia.

There has been considerable research using unsupervised machine learning methodologies in medical sciences not limited to psychiatry. One study reviewed the literature on detecting various diseases via computer-aided diagnosis and identified the best machine learning methodology for each disease [7]. Specific to using images as inputs, researchers have developed a single-layer sparse autoencoder to automatically classify tissue types from dynamic contrast-enhanced magnetic resonance imaging [8]. Another study discovered ground-truth networks in the brain through unsupervised learning of functional magnetic resonance imaging data [9]. As these research projects demonstrate, unsupervised learning has shown its potential to produce highly accurate models in the real world.

Several approaches have been proposed to handle the unstructured data type common in medical research. The support vector machine algorithm has been used on brain images for detecting Alzheimer disease in patients as well as for finding related brain parts with signs of Alzheimer disease [10]. One study built a convolutional neural network model to learn relevant features from unstructured raw data automatically and then made a convolutional neural network–based multimodal disease risk prediction model [11]. Another work collected data from an online social network (Twitter), constructed symptom weighting vectors by exploiting sentiment analysis, and tried to detect latent infectious diseases [12]. Some researchers have adopted wearable devices as inputs and built a neural network based on a multilayered perceptron to detect cardiovascular disease [13].

Despite the potential of a precision approach to insomnia treatment, little effort has been made to classify patients with insomnia using sleep and behavioral patterns. We hypothesized that certain symptom clusters representing specific sleep and/or behavioral patterns exist among individuals with insomnia. Sleep and behavioral characteristics that can affect insomnia symptoms are easily collected through smart bands. In this study, we tried to find certain clusters representing different sleep or behavioral characteristics using smart band data. Our unsupervised learning classification approach can be a cost-effective alternative that can bring positive insights for developing everyday interventions to assist in insomnia recovery (see Multimedia Appendix 1 for more information about implementation details including codes and datasets).

## Methods

### Recruitment

We applied unsupervised learning to gathered time-series data from an experiment. Participants were recruited using an online board of a large university community. To recruit subjects with insomnia symptoms, we used the Insomnia Severity Index (ISI) [14], Korean version [15]. The ISI is calculated based on responses to 7 questions that ask about sleep problems individuals have experienced in the most recent 2 weeks. For example, the questionnaire asks “How satisfied/dissatisfied are you with your current sleep pattern?” and the respondents answer using a 5-point Likert scale (1=very satisfied, 2=satisfied, 3=moderately satisfied, 4=dissatisfied, 5=very dissatisfied). This index is used as a standard metric of treatment response in clinical research.

A total of 50 participants whose ISI scores were 15 or above (ie, an indication of a mild to severe level of insomnia) participated (26 male; average age 22.63 [SD 3.02] years). Height and weight information were also gathered to calculate body mass index (BMI). In the study, we recruited young, healthy participants. Applicants being treated for medical or psychiatric illnesses were excluded from the experiment. The experiment was approved by the Korea Advanced Institute of Science and Technology institutional review board (approval number KH2018-40). Subjects’ behavioral and sleep characteristics were collected via a smart band, the Charge 2 (Fitbit Inc) [16].

### Intervention

**Figure 1:**
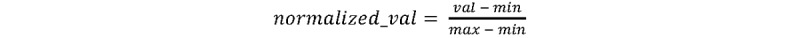
Min-max scaler for normalizing each feature, where all values for each feature are normalized between 0 and 1.

The experiment was conducted for 6 weeks from April 23 to June 3, 2018, and the subjects’ data were sent to a server. The first author gave weekly reminders to encourage subjects to wear the device continuously. Eight subjects were excluded from the study since they failed to wear the device for longer than 2 consecutive days. The remaining 42 participants finished our experiment, and we analyzed data from the subjects (19 male; average age 22.00 [SD 2.79]). The data features gathered and analyzed in our study are shown in Textbox 1. All values were normalized before analysis (see Figure 1 for the normalization method).

**List of data gathered from the smart band–wearing experiment.**

Modality 1: Sleep pattern (source: Fitbit)

- sleep_start_time: time when a user goes to bed in units of seconds
- sleep_end_time: time when a user gets out of bed
- onbed_min: total time duration for staying in bed
- sleep_min: total time duration for actual sleeping
- sleep_efficiency: sleep min/onbed min
- awaken_min: total time duration of waking while sleeping
- awaken_moments: total frequency of waking up while sleeping

Modality 2: Daily activity (Fitbit)

- calories_consumed: total number of calories consumed per day
- active_calories: total number of calories consumed for activities per day
- Walks: total frequency of steps per day
- distance: total distance a user moves per day
- Stairs: total frequency at which a user climbs stairs per day
- active_ratio: daily moving time over the total time wearing the device

Modality 3: Personal demographic (Survey)

- Age: age of the participant
- Gender: gender of the participant
- Body mass index: kg/m2
- Insomnia Severity Index: result from the survey

Fitbit provides a rich set of information about the wearer’s sleep. For example, it tracks the total time that a person is in bed as well as the predicted total sleep time. Its autodetection and prediction are based on various behavioral or biological patterns such as information on heart rate and movement. There is one paper confirming the validity of the data from the Charge 2 [17]; it reports that sleep sensitivity (ie, predicting falling asleep) of Fitbit is remarkable, about 0.96, and the measured wake after sleep onset is similar to that of the existing polysomnography (PSG) method. Another study also demonstrated that the retrieved data from actigraphy corresponded acceptably well to that of PSG [18]. However, at the same time, we need to be cautious of using the data retrieved from wearables, since there might be a specificity (relatively low precision) issue with the wearables [19]. While it is possible for smart bands to give false sleep reports, we chose smart bands over PSG because they are easier to use and can obtain sleep measures regularly not in the clinical setting but in the wild. In addition to the sleep log, the Fitbit also reports a variety of information about the subjects’ activity, including the calories consumed, steps walked, distance moved, and stairs climbed.

### Data Analysis

Among the Fitbit-provided features in Textbox 1, we considered the reliability of information about sleep stages to be relatively low; therefore, 3 sleep features (rapid eye movement sleep, narrow sleep, deep sleep) were excluded from the analysis. Instead, sleep efficiency was added, which is calculated as the fraction of time dedicated to actual sleep out of the time spent lying in bed. Sleep efficiency is an essential factor in determining sleep quality [20]. Additionally, we introduced a new variable, the active ratio, which examines the total fraction of time spent on any activity.

Due to the nature of sleep, we needed to examine time-series data and consider the entire sequence of activities leading to each sleep event [21]. Today’s sleep can be affected collectively by today’s activity, yesterday’s sleep, and so on. Therefore, it may be acceptable to jointly consider logs like this across multiple sleep and behavioral features. Our goal was to automatically identify clusters of sleep and behavioral features in hopes of identifying meaningful groups of individuals with insomnia who exhibit similar sleep-related dysfunctions.

## Results

### Approach Overview of Cluster Analysis

Cluster analysis has been a popular research topic in computer science [22]. In this study, we have tried two different approaches to clustering: synchronic and diachronic. In the synchronic approach, we would consider the dataset as one whole snapshot and cluster features within a snapshot all together. There were two issues with the synchronic approach (see Multimedia Appendix 1 for more detailed research procedures and results of this approach): (1) the clustering performance was not good enough in general and (2) this approach does not consider the consecutive patterns within and between features. Thus, we developed a novel diachronic approach, presented in the following section, in which we cluster the chunk consecutive daily logs first and then classify users based on their dominant clusters of chunk logs. This method represents insomnia-related patterns as a sequence of images.

### Diachronic Unsupervised Learning of Insomnia Patterns

Supervised learning of clinical data may develop an overfitting problem because data are unstructured, sparse, noisy, or irregular [23]. In such conditions, unsupervised learning can be a good alternative. In particular, a convolutional autoencoder (CAE) that learns the abstract latent representations of data is known to be appropriate for the task [24]. We implemented a CAE and prepared a collage of daily features as an input image. This approach allowed us to capture the entire set of features simultaneously within the assigned time window.

When clustering high-dimensional data, it is crucial to reduce dimensions to avoid expensive computational costs and memory loss [25]. In handling high-dimensional data, the target clusters often lie in subspaces of the full space [26]. Hence, reducing dimensions via conventional methods such as singular value decomposition or principal component analysis may not yield correct clustering results, mainly when the target clusters are not in the same subspace. To avoid this issue, we implemented a CAE to effectively reduce the dimensions in the data since the latent variables of the CAE may include subspace information via filters. We also chose to implement CAE before clustering because a previous study argued that subsequence time-series clustering based on a sliding window might not be meaningful [27]. By treating the data with CAE first and then applying to cluster, the outcome patterns of the clusters are affected not by the subsequence time-series data but by the derived latent variables per image. Moreover, we can also expect that by presuming the data into images, the latent variables of CAE are more robust to the noisy daily changes of the Fitbit logs.

### Clustering Steps

The neural net–based clustering approach operates in 5 steps.

#### Step 1: Preprocessing of Time-Series Data

We combine modality 1 (sleep pattern) and modality 2 (daily activity) in Textbox 1 to construct a multiplex vector of 12 dimensions (cf, modality 3, personal demographics, was not included since its data did not frequently change over the 6-week period, so it was solely employed in the comprehensive qualitative analysis process). In this step, with respect to modality 1, the *onbed_min* feature was excluded from the source dataset because it showed high correlations with other features, similar to the synchronic approach (see Multimedia Appendix 2 for the cross-correlations among 12 features of modalities 1 and 2). Let sleep vector **v_s_**=*{x*1, *x*2, ..., *x*8*}* and activity vector **v_a_**=*{y*1, *y*2, ..., *y*6*}*; then, the daily multiplex vector of subject *α* **v**_α_=*{x*1_α_, ..., *x*8_α_, *y*1_α_, ..., *y*6_α_}.

#### Step 2: Composing Sequential Images From Data

**Figure 2:**
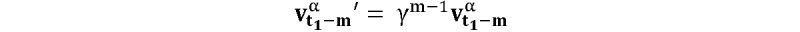
Consecutive multiplex vector of the subject α per day on the 1st image chunk, where t_1_ is defined as the 9th of v^α^ (ie, the 9th day from the starting date) and m as the corresponding closeness rank number (m^th^ of v^α^) to t_1_ within the image chunk (1≤m≤8).

Sequentially connected multiplex vectors can be treated as an image chunk. We then composed the consecutive multiplex matrix to learn latent variables from images that represent individuals’ daily log series. We modeled the daily sleep patterns as a Markov decision process because, according to previous sleep research, sleep deprivation (ie, sleep debt) has cumulative effects on waking function, and tonight’s sleep debt will mostly be affected by recent sleep patterns (ie, sleep 1 to 2 days before) [28]. As a result, we connected each daily multiplex vector with a sliding window over 8 days (ie, the total 8-day logs are combined as one window always to include a weekend, where a window slides to the next vector, which is the next day). We applied a discount factor (γ) such that on the first window, the consecutive multiplex vector of the subject α per day can be formulated as in Figure 2.

**Figure 3:**
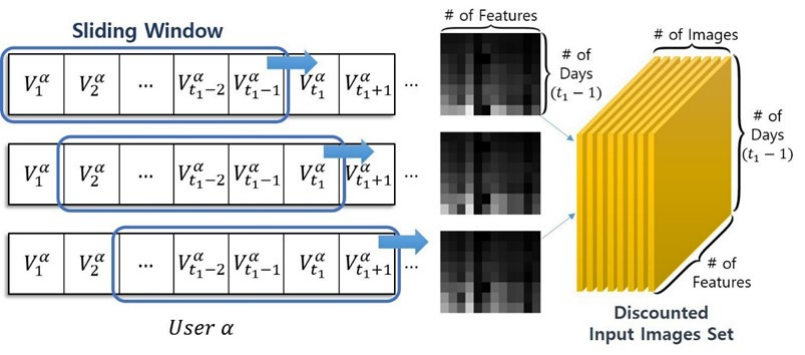
Converting the consecutive sleep and activity data to images: sliding windows of 8-day chunks (# of Features=12, # of Days [ie, win-size]=8).

**Figure 4:**
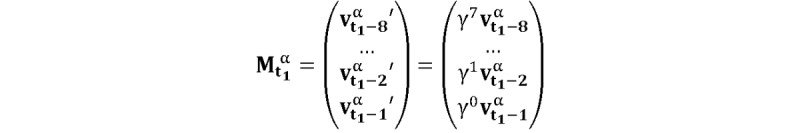
Sequential multiplex matrix of the subject α on the 1st image chunk, where t_1_=9.

**Figure 5:**
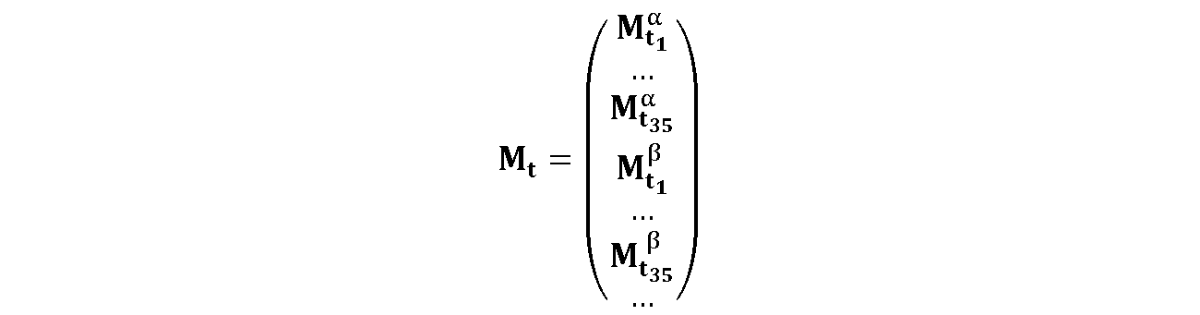
Sequential multiplex matrix of all subjects for all 35 images, where β indicates the 2nd participant, t_n_≤43, and t_n+1_=t_n_+1.

We then finalized the sequential multiplex matrix, as shown in Figure 3. Fixing 8 days as a window size leaves a total of 42 days worth of data (ie, 42 data points). This result means that each user is represented by 35 sets of images (42–8+1=35), which is the batch size. The total number of input image chunks for the CAE is 1470 (42 users×35 images=1470). Let the sequential multiplex matrix of the first image chunk for the subject α be defined as in Figure 4. As a consequence, the sequential multiplex matrix of all subjects for all 35 images can be framed as in Figure 5 (see Multimedia Appendix 1 for more information about the mathematical notations).

#### Step 3: Learning Representations With Reducing Dimensions via Convolutional Autoencoder

**Figure 6:**
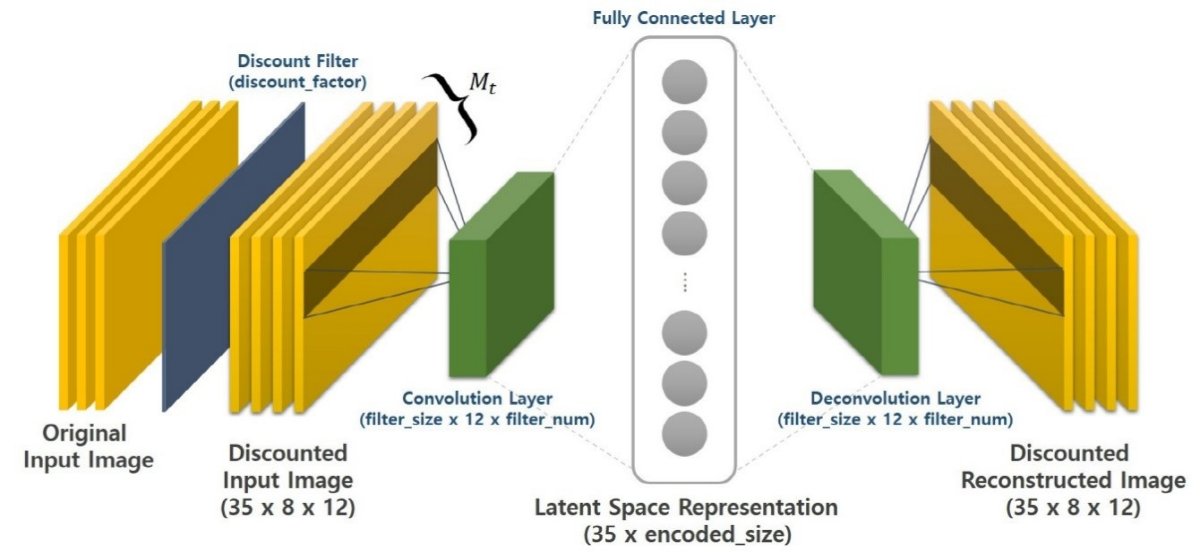
Convolutional autoencoder to find the latent variables of insomnia-related patterns per 8 days.

In the proposed model depicted in Figure 6 for the designed CAE, the pooling layer has been omitted due to the small size of the input images (8×12). Additionally, we set up only one convolutional layer due to the relatively small amount of data (141,120 pixels=1470×8×12). Discount factor γ was set between 0.60 and 0.99. Then we iterated the model until finding the optimal learning rate, optimal γ value, optimal vertical size (cf, the horizontal size was fixed to 12, ie, the number of features), number of convolutional filters, and number of latent variables (ie, *encoded_size* in Figure 6).

**Figure 7:**
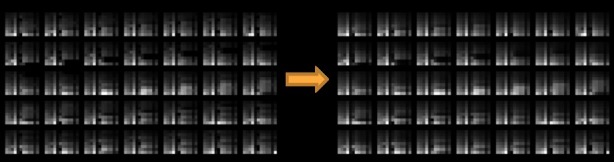
Convolutional autoencoder (CAE) reconstruction results of the learned CAE for one random subject.

We tested all combinations among 5 hyperparameters and found that the optimal values were 1e^–4^ with the AdamOptimizer (optimal learning rate); 0.75 (optimal γ value); 3 (optimal vertical size); 30 (number of convolutional filters); and 15 (number of latent variables), for each of these hyperparameters with the lowest L2-norm regularized reconstruction loss value of 1.09 (see Multimedia Appendix 1 for more information about the optimization and overfitting issue). To confirm the learning results, Figure 7 depicts the 35 input images and 35 reconstructed outputs of one random participant (*UserId* 26). This visual coherence ensures that the CAE efficiently reduced the input size dimensions (ie, 8×12→15) by learning the vital latent representations of data.

#### Step 4: Clustering Images via Latent Variables

**Figure 8:**
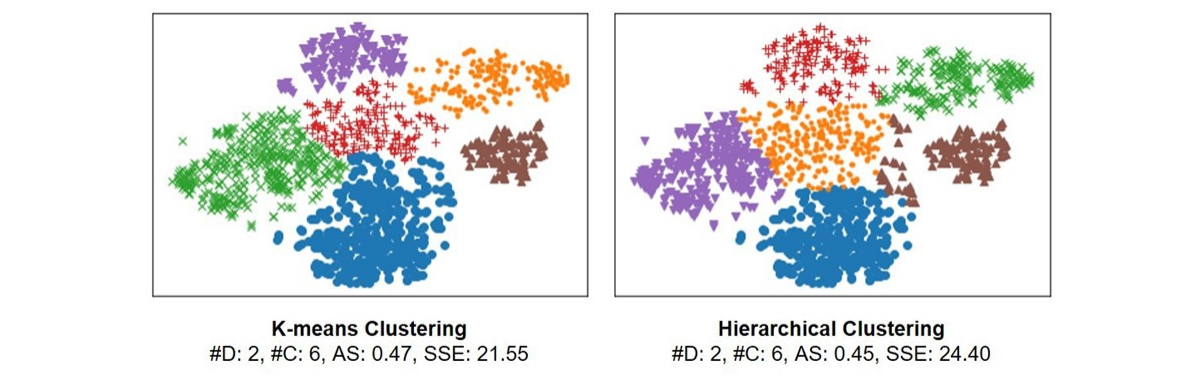
Clusters of 8-day chunk images based on sequential sleep/activity patterns. D: dimensions; C: clusters; AS: average silhouettes; SSE: sum of squared errors.

We clustered the latent variables as follows. As an initial step, we used t-SNE (ie, stochastic neighbor embedding with t-distribution) to reduce the number of dimensions from 15 to 2 additionally (ie, major hyperparameters for t-SNE were perplexity=30.0 [29,30], see Multimedia Appendix 1 for more information related to t-SNE; metric=euclidean; method=barnes_hut; the clustering result from the largest value of average silhouettes [AS] was chosen out of 100 trials). We then applied k-means and hierarchical clustering algorithms to form clusters of the 8-day chunk images. The results are displayed in Figure 8; both clustering results indicate the same cluster number of 6, and they were found reasonable by the AS values. However, the results of k-means clustering showed better results following the AS as well as the sum of squared errors (SSE). Therefore, we chose k-means to be the choice of the final user clustering step.

#### Step 5: Clustering Individuals With Insomnia

Each cluster of the 8-day images was labeled as cluster A, B, C, D, E, and F. We then composed a sequence of codes for each user (eg, the best-matched cluster code for each of the 35 images representing the user’s logs, such as “*A-A-D-C-C-E-B-...*”). Finally, to determine one dominant cluster type per user, the PageRank algorithm was implemented and used among the stochastic relations between those 6 extracted labels. This intuition is feasible since participants’ sequential codes, which are the series of the latent space representations per 8-day image for both sleep pattern and daily activity modalities, became the subjects’ series of the comprehensive insomnia-related patterns; therefore, the codes can be modeled as a hidden Markov model, meaning that one’s next state is dependent on the current state [28]. We then set the largest stochastic value of the labels per participant to be the dominant cluster, which is identical to finding a cluster with the highest PageRank value (ie, the probability of arriving at the cluster after many steps).

### Evaluation Outcomes

Table 1 shows the clustering evaluation results among the two approaches concerning the diachronic clustering of daily vectors (**v**). The first approach is without CAE, meaning the original dataset is converted into chunk images (one image is composed of the 8 consecutive daily vectors with 96 dimensions) and then filtered via t-SNE (96→2 dimensions) and clustered by 3 clustering methods. The second approach is our model, and as introduced above, the original dataset is converted into chunk images and then inputted into CAE, and the latent variables of CAE are clustered by 3 clustering methods after the variables are filtered through t-SNE (96→15→2 dimensions).

We calculated two metrics to measure the clustering performance: the AS and the SSE. With AS, if the value is greater or equal to 0.4, then the clustering result is guaranteed to be significant [31]. By contemplating consecutive pattern information together via composing 35 images from the weekly based consecutive **v** per user, we could confirm that k-means clustering with our process showed the best performance (ie, the highest value of AS and the lowest of SSE) in clustering **v**. With respect to the first approach, the clustering performances were worse, and the possible reason for this result is that although the number of dimensions becomes large due to the chunked **v**, the approach could not successfully extract the latent features of the chunks while dramatically reducing the number of dimensions from 96 to 2.

Table 2 presents how the 42 subjects were eventually divided across the identified clusters of **v** by using our process with k-means clustering. Cluster F did not contain any members. The table shows the PageRank value, which represents the strength of the association between subjects and clusters. Cluster IDs are sorted by this PageRank value. Both cluster A and cluster D have the largest subject count of 12 people. The average BMI and ISI values are also given for each of the clusters. The BMI and ISI values were similar across clusters.

**Figure 9:**
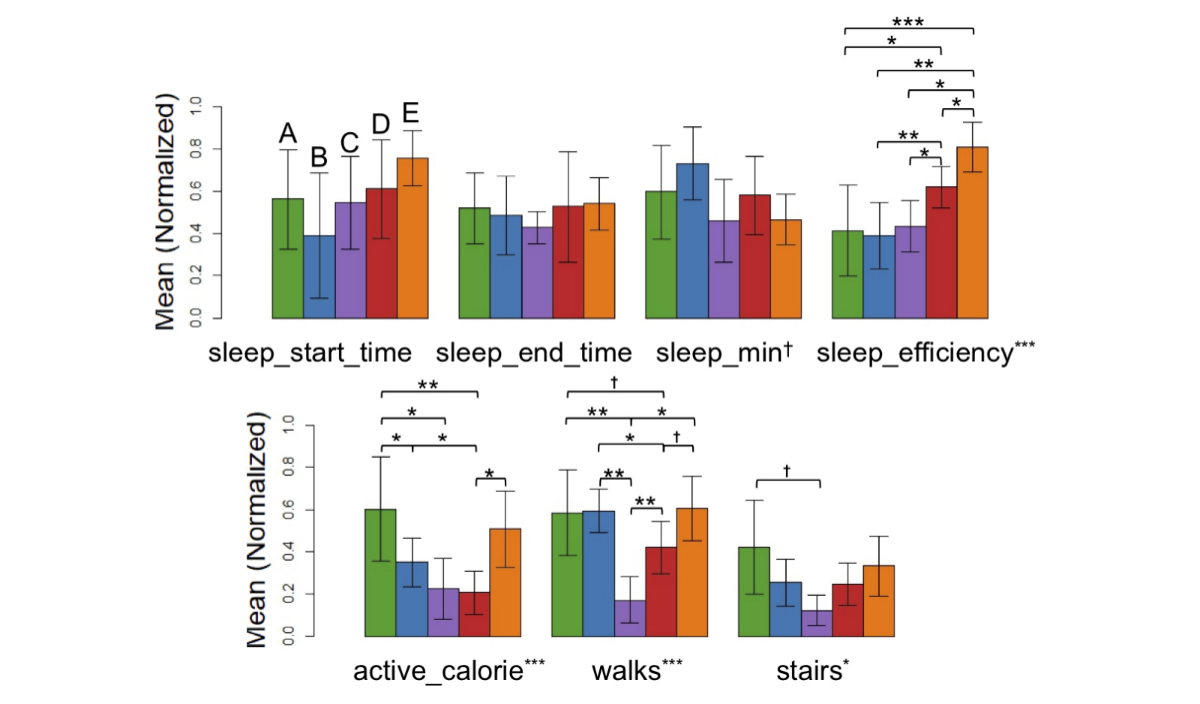
Bar plots of the major smartband features per cluster (^†^*P*<.10, *P<.05, **P<.01, ***P<.001 in analysis of variance on rank and Kruskal-Wallis test results for 5 groups, also find Tables MA3-a and MA3-b in Multimedia Appendix 3).

A psychiatrist conducted both qualitative and quantitative analyses of the 5 clusters. The number of subjects for each cluster is small and does not meet normality, and therefore, we performed an analysis of variance (ANOVA) on rank and Kruskal-Wallis test. There were several features that showed statistically significant differences among clusters (ANOVA on rank: F_4,37_=2.36, *P*=.07 for *sleep_min*; F_4,37_=9.05, *P*<.001 for *sleep_efficiency*; F_4,37_=8.16, *P*<.001 for *active_calorie*; F_4,37_=6.53, *P*<.001 for *walks*; F_4,37_=3.51, *P*=.02 for *stairs*). Detailed results were described in Figure 9 as well as Tables MA3-a and MA3-b in Multimedia Appendix 3 (we also performed the ANOVA and post hoc Tukey honest significant difference test and included the results in the same supplementary material for reference).

**Table 1:** Evaluation of user clustering results for two diachronic approaches.

Approach	NC^a^	AS^b^	SSE^c^	NEO^d^	Remark
First with DBSCAN^e^	1	0.2075	119.4467	14	Chunk images used without a CAE^f^
First with hierarchical clustering	4	0.3394	35.0190	—^g^	Chunk images used without a CAE
First with k-means clustering	5	0.3941	22.6103	—	Chunk images used without a CAE
Ours with DBSCAN	4	0.1275	64.7179	30	Chunk images used with a CAE
Ours with hierarchical clustering	6	0.4485	24.3988	—	Chunk images used with a CAE
Ours with k-means clustering	6	0.4653	21.5532	—	Chunk images used with a CAE

^a^NC: number of clusters.

^b^AS: average silhouettes.

^c^SSE: sum of squared errors.

^d^NEO: number of excluded outliers.

^e^DBSCAN: density-based spatial clustering of applications with noise.

^f^CAE: convolutional autoencoder.

^g^Not applicable.

**Table 2:** Diachronic clustering results of 42 participants suffering from insomnia.

Cluster ID	A	B	C	D	E	F
Subjects, n (%)	12 (29)	9 (21)	4 (10)	12 (29)	5 (12)	0 (0)
PageRank	0.57	0.54	0.45	0.42	0.36	—^a^
BMI^b^ (kg/m^2^), mean (SD)	22.8 (3.3)	20.8 (2.3)	26.0 (2.7)	20.2 (3.0)	22.9 (2.3)	—
ISI^c^, mean (SD)	19.1 (1.5)	19.1 (2.4)	18.2 (1.6)	16.9 (1.8)	18.8 (2.4)	—

^a^Not applicable.

^b^BMI: body mass index.

^c^ISI: Insomnia Severity Index.

## Discussion

### Principal Findings

This research conducted a 6-week experiment to collect smart band data and analyzed it to identify relationships between insomnia and daily activities. Our analysis finds that participants who seemingly face similar levels of insomnia based on the ISI score belonged to different clusters based on unsupervised learning. This finding means that our neural net–based clustering method could identify, beyond conventional diagnosis, new meaningful sleep-activity relationships that could be used to devise tailored interventions. Our method could determine which cluster an individual belongs to via data indicators of sleep and behavior acquired in the study.

Among the derived clusters of people with insomnia (see Figure 9), we found clusters *B*, *C*, and *E* to be more similar to one another and clusters *A* and *D* to contain similar sleep-activity profiles. Within the former group, subjects in cluster *B* were relatively long sleepers (even with insomnia) with low sleep efficiency. These subjects were in bed long hours, yet they could not easily fall asleep. Sleep restriction was a potentially useful intervention for these subjects [32]. In contrast, the subjects in cluster *E* exhibited a short sleep time with high sleep efficiency. These subjects also burned more calories per day. Chronic restriction of sleep may have affected symptoms of insomnia in this group [33]. While subjects in cluster *C* seemed to be morning persons, as indicated by the awake time patterns, their activity levels were relatively low, as shown in the active calorie, walk, and stair data. These subjects were treated with different interventions from the other similar clusters. In the latter group, clusters *A* and *D* had similar sleep patterns, such as total sleep time and sleep efficiency. However, subjects in cluster *A* indicated higher activity levels, such as walks and stairs, compared with cluster *D*. Cluster *D* also showed the lowest ranges for both BMI and ISI compared with other clusters, where a smaller BMI value represents nonobese status and a smaller ISI value represents a milder insomnia level. Such subtle differences would have been difficult to notice with conventional methods or supervised learning. These results suggest that cluster-based intervention for individuals with insomnia could be an accurate method for alleviating symptoms of insomnia.

This study demonstrates that unsupervised learning of insomnia activity data gathered from wearable fitness devices could identify meaningful clusters. The 5 derived clusters each had distinctive characteristics, meaning that they can be used to derive different therapies and diagnoses. In accordance with our finding, one study also classified thousands of participants who answered online surveys by using latent class analysis so that they could find insomnia disorder subtypes [34], yet they were more focused not on past behavioral and sleeping patterns but on individual personalities as well as stressful surroundings in revealing the clusters. One more difference is that they solely used a conventional statistical method. Our study highlights that merely looking at sleep logs can reveal a limited aspect of insomnia; one needs to look at the full spectrum of both daily activities and sleep logs. Finally, our findings align with those of a previous study that revealed the average sleep quality is better related to many behavioral aspects of the person than the average sleep quantity [35].

### Limitations

This work has several limitations. First, participants were from the same university, which may induce sampling biases. A larger scale study can draw conclusions that apply to the general public. In the future, we plan to conduct experiments targeting generic patients who visit hospitals regularly due to insomnia to ensure the generality of the clustering result. Second, a small sample size can reduce the statistical power, and it can affect the reducibility for future study. Third, various behaviors, such as consuming alcohol, tobacco, or caffeine, can affect patterns of sleep and activity. However, these factors were not considered in the current clustering analyses, mainly due to the small sample size and limited information on these behaviors (eg, consuming frequency per day or week). In that sense, a future study is needed to verify our preliminary insights. Fourth, when constructing the CAE, we prepared the input and output images irrespective of subjects. As a consequence, the learned latent variables cannot capture subtle dissimilarities that may exist across participants. To reduce this potential bias, a learning model may diminish the latent factors in composing images among participants while training. Last, we used t-SNE when reducing the dimensionality of the latent features of CAE images, and there could be type 1 errors when clustering dimension-reduced data because t-SNE forcibly fits original data into a t-distribution. We further plan to adopt and test other dimensionality reduction methods toward the extracted latent variables of CAE images to relax the possible type 1 error issue.

### Conclusion

This study, although preliminary, gives new insights for future studies in the field of mobile health. Motivated by the results of this study, in the future we hope to develop tailored intervention strategies that can be matched to each cluster for relieving insomnia symptoms, which will be a meaningful step in precision psychiatry. In the meantime, we plan to consider qualitative factors such as the level of sleep as well as quantitative aspects of sleep-related factors.

## Appendix

Multimedia Appendix 1 Description of the datasets and the codes utilized at the current research and detailed procedure of user clustering based on the synchronic approach and its evaluation outcomes with additional descriptions on the clustering steps of the diachronic unsupervised learning approach.

Multimedia Appendix 2 Cross rank correlation matrix among 12 features of modality 1 and 2 used in clustering, regardless of the derived cluster ID (N=42) and with regard to each derived cluster ID (# of clusters=5).

Multimedia Appendix 3 Test results with respect to sleep pattern-related (modality 1) and daily activity-related (modality 2) features among 5 derived clusters of insomnia sufferers: (1) analysis of variance on rank and Kruskal-Wallis and (2) analysis of variance and post hoc Turkey honest significant difference.

## Abbreviations

ANOVA: analysis of variance
AS: average silhouettes
BMI: body mass index
CAE: convolutional autoencoder
ISI: Insomnia Severity Index
PSG: polysomnography
SSE: sum of squared errors
t-SNE: t-distributed stochastic neighbor embedding
