# Supplementary material for: Clustering Insomnia Patterns by Data From Wearable Devices: Algorithm Development and Validation Study
Source: JMIR Mhealth Uhealth. 2019 Dec 5;7(12):e14473. doi: 10.2196/14473 (PMC6923760; doi:10.2196/14473)
Supplement: Multimedia Appendix 1 [file mhealth_v7i12e14473_app1.pdf]

Multimedia Appendix 1

Data and Code Description

We have released the datasets and the codes utilized at the current research[1]. About the data, we provide two types of dataset: raw and latent variables from CAE (convolutional autoencoder). The "raw" dataset means the original dataset retrieved from the experiment. The features included in the dataset are as follows:

- userId: each subject's unique ID after the pre-processing of anonymization (total 42 user)
- month: month of the generated data
- date: date of the generated data
- sleep_start_time: time when a user goes to bed in units of seconds (for the longest sleep time among all sleep times per day)
- sleep_end_time: time when a user gets out of bed
- sleep_min: total time duration for actual sleeping
- sleep_efficiency: sleep_min / onbed_min (c.f., onbed_min is a total time duration for staying in bed)
- awaken_min: total time duration of waking while sleeping
- awaken_moments: total frequency of waking up while sleeping
- nap_count: total frequency of nap times (i.e., excluding the longest sleep time) per day
- total_nap_min: total time duration for the accumulated actual nap times per day
- cal_consume: total number of calories consumed per day
- active_cal: total number of calories consumed for activities per day
- walks: total frequency of steps per day
- distance: total distance a user moves per day
- stairs: total frequency at which a user climbs stairs per day
- active_ratio: daily moving time over the total time wearing the device

In addition, the "latent variables from CAE" dataset means the dataset of latent variables extracted via a CAE learning latent representations with reducing Dimensions. The features included in the dataset are as follows:

- imageId: each image's unique ID (c.f., image: a chunk composed by consecutive 8 daily multiplex feature vectors)
- latent variables: extracted with 15 dimensions (i.e., # of features is 15) via CAE

About the code, we provide codes from *Step 1* to *Step 3* on the "Clustering Steps" section at the manuscript. Based on the codes, it is possible to imagify the given original data then to extract latent variables via CAE. For the remaining *Step 4* and *Step 5*, we have not included the corresponding codes since we also use the open sources of t-SNE and various conventional clustering methods. All codes have been tested running under Python 3.6.6 with the following packages installed (along with their dependencies): numpy = 1.16.0; pandas = 0.23.4; tensorFlow = 1.12.0; scikit-learn = 0.20.2.

---

[1] Implementation details including datasets and codes are available via the following web link: https://github.com/dscig/sleeps_clustering.

User Clustering Based on Synchronic Approach

We applied two basic clustering algorithms, k-means and hierarchical clustering, to the 12 features in Table 1 at the main script (c.f., onbed_min was excluded from clustering because it is highly correlated with other features in Modality 1, and the four features in Modality 3 in Table 1 were not utilized in clustering because we posit that these features were steady across the experiment period). In particular, after preprocessing the collected data, we tried to cluster individuals with insomnia based on two synchronic approaches and to cluster the daily vectors (**v**) of the participants based on the following synchronic approaches. The first approach clustered based on the whole dataset (42 days × 12 features = 504 dimensions per user) after filtering through t-SNE (t-distributed stochastic neighbor embedding) to reduce dimensionality (504 → 2), while the second approach clustered based on the parametric dataset using the mean and standard deviation (SD) values of each feature (24 features = 24 dimensions per user), meaning 42 days per user was considered one abstracted snapshot data after filtering through t-SNE (24 → 2). The third approach clustered not users but **v**: the dimensionality of the original dataset was directly reduced via t-SNE and then clustered by two clustering methods (i.e., **v** with 12 dimensions was directly reduced to 2 dimensions via t-SNE and then clustered: 12 → 2).

Evaluation Outcomes of User Clustering Based on Synchronic Approach

Table MA1 presents the clustering evaluation results of the three explained approaches. The AS values of the first and second approaches are less than 0.4, and the SSE values are relatively large considering the 42 clustered targets together. Therefore, we conclude that the clustering result is not significant and that it is not sufficient to directly cluster users based on their daily Fitbit logs.

Additionally, k-means clustering for the third approach showed the best clustering performance (i.e., the highest value of average silhouette and a relatively low SSE collectively considering 1,470 clustered targets). However, the third approach is based on the original **v**, and therefore, the derived clusters cannot capture the daily and/or weekly based 'sequential patterns' for each user. In other words, when adopting the third approach, a total of 1,470 **v** are actually assumed to belong to the same time-period (day), which is not a correct manifestation of the real relations among **v**.

Table MA1. Evaluation of user clustering results for three synchronic approaches (†NC: number of clusters, AS: average silhouettes, SSE: sum of squared errors).

Approach	NC†	AS†	SSE†	Remark
1st w/ hierarchical clustering	6	0.2568	0.8198	Individual insomnia sufferers are clustered
1st w/ k-means clustering	4	0.3256	1.1103	
2nd w/ hierarchical clustering	4	0.3058	0.8589	Individual insomnia sufferers are clustered
2nd w/ k-means clustering	4	0.3240	0.7559	
3rd w/ hierarchical clustering	4	0.4880	22.3503	Daily vectors (**v**) are clustered
3rd w/ k-means clustering	4	0.5082	20.9306	

Additional Descriptions on Clustering Steps of Diachronic Unsupervised Learning Approach

Step 2. Composing Sequential Images from Data:

The notation t is related to the sequential label of the daily multiplex vector **v**, and in particular, the notation t_n indicates the first label of **v** on the next imagified 8-day chunk, in the case of the nth chunk. For instance, $t_1 = 9$, meaning that in the case of the first ($n = 1$) chunk, the first label of **v** on the next chunk is 9. In accordance with this description, $t_{35} = 43$, meaning that in the case of the 35th ($n = 35$) chunk, the first label of **v** on the next chunk is 43. Since we have made the chunks up until the 35th one, the upper limit of t_n becomes 43 ($t_n \leq 43$).

Step 3. Learning Representations with Reducing Dimensions via CAE:

About the "reconstruction error" of the model, which is the objective function of CAE, we use L2-norm regularized reconstruction loss (L) between the original image (y) and the reconstructed image ($\hat{y}$) for 1,470 images ($n = 1470$). The loss function can be formulated as follows:

$$L(y - \hat{y}) = \sum_{i=1}^{n} (y - \hat{y_i})^2 \; where \; n = 1470$$

In addition, with respect to the optimization and Overfitting issue, there are three points to address. First, since it is unsupervised learning, there is no label (Y) on the data. In that sense, we decide it is not needed to split the dataset into a training and a testing set. Second, when training the convolutional autoencoder, we tuned the hyperparameters not based on the average value of silhouette (AS) but based on the "reconstruction error" of the model. Therefore, we believe overfitting may not be the matter to concern much in our unsupervised model setting. Last, the main objective of the current work was to find optimal clusters on the given dataset from one particular experiment. We do not claim that the derived clusters shall be considered as general insomnia disorder subtypes.

Step 4. Clustering Images via Latent Variables:

We did not test all combinations of parameter values of t-SNE, and we recognized the changes in dimensions and perplexity can result in different clustering results. However, in the case of perplexity, the original paper proposing t-SNE suggested that the performance of SNE is fairly robust if its value is between 5 to 50 [1, 2]. Therefore, we have chosen perplexity as "30", and the overall tendency of clustering results would be reasonably consistent.

In addition, we used t-SNE for dimensionality reduction because of its "non-linearity" trait. Even if other methods such as PCA are easier and have no hyperparameter that needs to be tuned, most of them are linear and only capture the components with largest variances in a dataset. We also tried and got clusters by utilizing PCA too, but PCA was too sensitive on outliers and we obtained the incongruent results; the points of the 8-day chunks were mostly concentrated on one specific cluster.

References

1. Maaten LVD, Hinton G. Visualizing data using t-SNE. Journal of machine learning research 2008 Nov;9:2579-2605.
2. Wattenberg M, Viégas F, Johnson I. How to use t-SNE effectively. Distill 2016;1.10:e2.

< end of document >
