# Supplementary material for: Clustering Insomnia Patterns by Data From Wearable Devices: Algorithm Development and Validation Study
Source: JMIR Mhealth Uhealth. 2019 Dec 5;7(12):e14473. doi: 10.2196/14473 (PMC6923760; doi:10.2196/14473)
Supplement: Multimedia Appendix 2 [file mhealth_v7i12e14473_app2.pdf]

Multimedia Appendix 2

We could try to understand the meaning of the clustering results presented in Table 4 and Figure 5, by comparing the correlations among features on all participants and the correlations on participants belong to specific clusters, derived by the proposed convolutional autoencoder method. For instance, by comparing Table MA1, Table MA1-a, and Table MA1-d, it may be possible to deduce the unique traits of Cluster ID “A” and “D” as follows.

Overall, the cross correlations of Cluster ID “A” and “D” seem similar. However, the correlation between *sleep_min* and *sleep_efficiency* of Cluster ID “A” was statistically significant whereas that of Cluster ID “D” was not, meaning the overall sleeping time highly affects sleep quality only on Cluster ID “A”. We could also find that the correlations between *sleep_start (end)_time* and *walks (distance)* are statistically significant only on Cluster ID “A”. It may imply that activity levels affect sleep pattern on Cluster ID “A”, and this explanation aligns with the interpretation on the clustering results, provided at the Discussion section of the main script.

Table MA2. Cross correlation matrix among all features (total 12 features from Modality 1 and Modality 2 used in clustering) regardless to the derived Cluster ID (N=42). We present the Spearman's rank correlation rho with *P*-value († $P<.1$, * $P<.05$, ** $P<.01$, *** $P<.001$).

Feature	sleep_ start_ time	sleep_ end_ time	sleep_ min	sleep_ efficiency	awaken_ min	awaken_ moments	calories_ consumed	active_ calories	walks	distance	stairs	active_ ratio
sleep_start_ time	-	0.50***	-0.41***	0.12***	-0.36***	-0.31***	0.04†	-0.004	-0.03	-0.02	0.01	-0.04†
sleep_end_ time	-	-	0.49***	0.05*	0.26***	0.23***	0.02	0.02	0.03	0.03	0.02	0.02
sleep_min	-	-	-	0.07**	0.55***	0.53***	-0.01	0.03	0.06*	0.06**	0.02	0.06*
sleep_ efficiency	-	-	-	-	-0.73***	-0.37***	0.03	0.02	0.04	0.04†	0.001	0.004
awaken_ min	-	-	-	-	-	0.70***	-0.04†	0.002	0.02	0.01	0.01	0.04†
awaken_ moments	-	-	-	-	-	-	-0.03	0.03	0.03	0.03	0.04	0.08**
calories_ consumed	-	-	-	-	-	-	-	0.80***	0.51***	0.57***	0.40***	0.42***
active_ calories	-	-	-	-	-	-	-	-	0.78***	0.81***	0.54***	0.80***
Walks	-	-	-	-	-	-	-	-	-	0.99***	0.66***	0.75***
Distance	-	-	-	-	-	-	-	-	-	-	0.67***	0.74***
Stairs	-	-	-	-	-	-	-	-	-	-	-	0.44***
active_ ratio	-	-	-	-	-	-	-	-	-	-	-	-

Table MA2-a. Cross correlation matrix among all features (total 12 features from Modality 1 and Modality 2 used in clustering) with regards to the derived Cluster ID “A” (N=12, see Figure 5 at the main script). We present the Spearman's rank correlation rho with *P*-value († *P*<.1, * *P*<.05, ** *P*<.01, *** *P*<.001).

Feature	sleep_ start_ time	sleep_ end_ time	sleep_ min	sleep_ efficiency	awaken_ min	awaken_ moments	calories_ consumed	active_ calories	walks	distance	stairs	active_ ratio
**sleep_start_ time**	-	0.52***	-0.40***	0.003	-0.28***	-0.20***	0.02	0.09*	0.12**	0.11*	0.15***	0.20***
**sleep_end_ time**	-	-	0.47***	0.06	0.28***	0.27***	0.02	0.05	0.11*	0.11*	0.06	0.13**
**sleep_min**	-	-	-	0.25***	0.44***	0.47***	-0.01	-0.06	-0.01	0.000	-0.09*	-0.06
**sleep_ efficiency**	-	-	-	-	-0.67***	-0.17***	0.08†	0.02	0.07	0.08†	-0.03	0.03
**awaken_ min**	-	-	-	-	-	0.56***	-0.06	-0.04	-0.05	-0.05	-0.03	-0.06
**awaken_ moments**	-	-	-	-	-	-	0.03	0.05	0.03	0.03	0.003	0.05
**calories_ consumed**	-	-	-	-	-	-	-	0.96***	0.84***	0.85***	0.52***	0.81***
**active_ calories**	-	-	-	-	-	-	-	-	0.89***	0.89***	0.59***	0.90***
**Walks**	-	-	-	-	-	-	-	-	-	0.998***	0.71***	0.82***
**Distance**	-	-	-	-	-	-	-	-	-	-	0.70***	0.81***
**Stairs**	-	-	-	-	-	-	-	-	-	-	-	0.53***
**active_ ratio**	-	-	-	-	-	-	-	-	-	-	-	-

Table MA2-b. Cross correlation matrix among all features (total 12 features from Modality 1 and Modality 2 used in clustering) with regards to the derived Cluster ID “B” (N=9, see Figure 5 at the main script). We present the Spearman's rank correlation rho with *P*-value († P<.1, * P<.05, ** P<.01, *** P<.001).

**Feature**	**sleep_ start_ time**	**sleep_ end_ time**	**sleep_ min**	**sleep_ efficiency**	**awaken_ min**	**awaken_ moments**	**calories_ consumed**	**active_ calories**	**walks**	**distance**	**stairs**	**active_ ratio**
**sleep_start_ time**	-	0.48^{***}	-0.52^{***}	0.13^{*}	-0.42^{***}	-0.49^{***}	$0.09^{\dagger}$	0.01	0.03	0.02	-0.03	-0.13^{**}
**sleep_end_ time**	-	-	0.42^{***}	0.13^{*}	0.19^{***}	$0.09^{\dagger}$	-0.07	-0.11^{*}	-0.05	-0.06	-0.05	-0.14^{**}
**sleep_min**	-	-	-	0.14^{**}	0.51^{***}	0.55^{***}	$-0.09^{\dagger}$	-0.05	-0.03	-0.03	-0.01	0.04
**sleep_ efficiency**	-	-	-	-	-0.71^{***}	-0.26^{***}	0.25^{***}	0.24^{***}	0.18^{***}	0.18^{***}	0.08	0.20^{***}
**awaken_ min**	-	-	-	-	-	0.63^{***}	-0.25^{***}	-0.21^{***}	-0.16^{**}	-0.16^{**}	-0.07	-0.16^{**}
**awaken_ moments**	-	-	-	-	-	-	-0.21^{***}	-0.11^{*}	$-0.10^{\dagger}$	$-0.10^{\dagger}$	0.05	-0.03
**calories_ consumed**	-	-	-	-	-	-	-	0.93^{***}	0.65^{***}	0.66^{***}	0.22^{***}	0.71^{***}
**active_ calories**	-	-	-	-	-	-	-	-	0.72^{***}	0.73^{***}	0.29^{***}	0.83^{***}
**Walks**	-	-	-	-	-	-	-	-	-	0.999^{***}	0.56^{***}	0.51^{***}
**Distance**	-	-	-	-	-	-	-	-	-	-	0.56^{***}	0.52^{***}
**Stairs**	-	-	-	-	-	-	-	-	-	-	-	0.17^{***}
**active_ ratio**	-	-	-	-	-	-	-	-	-	-	-	-

Table MA2-c. Cross correlation matrix among all features (total 12 features from Modality 1 and Modality 2 used in clustering) with regards to the derived Cluster ID "C" (N=4, see Figure 5 at the main script). We present the Spearman's rank correlation rho with *P*-value († *P*<.1, * *P*<.05, ** *P*<.01, *** *P*<.001).

Feature	sleep_ start_ time	sleep_ end_ time	sleep_ min	sleep_ efficiency	awaken_ min	awaken_ moments	calories_ consumed	active_ calories	walks	distance	stairs	active_ ratio
**sleep_start_ time**	-	0.62***	-0.35***	0.11	-0.33***	-0.30***	-0.11	-0.07	-0.08	-0.09	-0.10	0.03
**sleep_end_ time**	-	-	0.39***	0.04	0.26***	0.29***	0.07	0.13	0.19*	0.19*	0.08	0.22**
**sleep_min**	-	-	-	0.01	0.73***	0.77***	0.21**	0.24**	0.32***	0.31***	0.21**	0.20*
**sleep_ efficiency**	-	-	-	-	-0.62***	-0.22**	0.17*	0.13	0.10	0.09	0.12	0.07
**awaken_ min**	-	-	-	-	-	0.76***	0.04	0.11	0.20*	0.19*	0.11	0.14†
**awaken_ moments**	-	-	-	-	-	-	0.11	0.19*	0.27***	0.26***	0.17*	0.18*
**calories_ consumed**	-	-	-	-	-	-	-	0.93***	0.79***	0.80***	0.68***	0.79***
**active_ calories**	-	-	-	-	-	-	-	-	0.88***	0.89***	0.75***	0.90***
**Walks**	-	-	-	-	-	-	-	-	-	0.998***	0.86***	0.79***
**Distance**	-	-	-	-	-	-	-	-	-	-	0.86***	0.79***
**Stairs**	-	-	-	-	-	-	-	-	-	-	-	0.68***
**active_ ratio**	-	-	-	-	-	-	-	-	-	-	-	-

Table MA2-d. Cross correlation matrix among all features (total 12 features from Modality 1 and Modality 2 used in clustering) with regards to the derived Cluster ID "D" (N=12, see Figure 5 at the main script). We present the Spearman's rank correlation rho with *P*-value († *P*<.1, * *P*<.05, ** *P*<.01, *** *P*<.001).

Feature	sleep_ start_ time	sleep_ end_ time	sleep_ min	sleep_ efficiency	awaken_ min	awaken_ moments	calories_ consumed	active_ calories	walks	distance	stairs	active_ ratio
**sleep_start_ time**	-	0.46***	-0.35***	-0.004	-0.26***	-0.14**	-0.09†	-0.11*	-0.06	-0.06	-0.02	-0.03
**sleep_end_ time**	-	-	0.58***	-0.06	0.43***	0.40***	0.02	-0.03	-0.02	-0.02	-0.04	0.02
**sleep_min**	-	-	-	0.01	0.67***	0.56***	0.09†	0.05	0.01	0.02	-0.001	0.05
**sleep_ efficiency**	-	-	-	-	-0.67***	-0.33***	0.06	0.03	-0.04	-0.03	-0.03	-0.001
**awaken_ min**	-	-	-	-	-	0.68***	0.01	0.01	0.03	0.04	0.03	0.03
**awaken_ moments**	-	-	-	-	-	-	-0.06	-0.04	-0.03	-0.03	-0.01	-0.005
**calories_ consumed**	-	-	-	-	-	-	-	0.90***	0.68***	0.71***	0.38***	0.69***
**active_ calories**	-	-	-	-	-	-	-	-	0.86***	0.87***	0.52***	0.87***
**Walks**	-	-	-	-	-	-	-	-	-	0.997***	0.67***	0.77***
**Distance**	-	-	-	-	-	-	-	-	-	-	0.67***	0.77***
**Stairs**	-	-	-	-	-	-	-	-	-	-	-	0.47***
**active_ ratio**	-	-	-	-	-	-	-	-	-	-	-	-

Table MA2-e. Cross correlation matrix among all features (total 12 features from Modality 1 and Modality 2 used in clustering) with regards to the derived Cluster ID “E” (N=5, see Figure 5 at the main script). We present the Spearman's rank correlation rho with *P*-value († *P*<.1, * *P*<.05, ** *P*<.01, *** *P*<.001).

Feature	sleep_ start_ time	sleep_ end_ time	sleep_ min	sleep_ efficiency	awaken_ min	awaken_ moments	calories_ consumed	active_ calories	walks	distance	stairs	active_ ratio
**sleep_start_ time**	-	0.49***	-0.31***	0.08	-0.26***	-0.18*	0.08	0.03	-0.06	-0.05	0.03	-0.03
**sleep_end_ time**	-	-	0.60***	0.06	0.26***	0.16*	0.13†	0.13†	0.03	0.04	0.14†	0.01
**sleep_min**	-	-	-	0.15*	0.40***	0.26***	0.07	0.10	0.08	0.09	0.14†	-0.01
**sleep_ efficiency**	-	-	-	-	-0.80***	-0.58***	-0.01	-0.07	0.04	0.04	0.01	-0.08
**awaken_ min**	-	-	-	-	-	0.73***	0.02	0.09	0.01	0.003	0.06	0.07
**awaken_ moments**	-	-	-	-	-	-	-0.12†	-0.02	-0.03	-0.04	0.01	0.03
**calories_ consumed**	-	-	-	-	-	-	-	0.66***	0.43***	0.49***	0.64***	0.35***
**active_ calories**	-	-	-	-	-	-	-	-	0.60***	0.62***	0.53***	0.83***
**Walks**	-	-	-	-	-	-	-	-	-	0.996***	0.53***	0.59***
**Distance**	-	-	-	-	-	-	-	-	-	-	0.56***	0.59***
**Stairs**	-	-	-	-	-	-	-	-	-	-	-	0.34***
**active_ ratio**	-	-	-	-	-	-	-	-	-	-	-	-

< end of document >
