# Supplementary material for: Clustering Insomnia Patterns by Data From Wearable Devices: Algorithm Development and Validation Study
Source: JMIR Mhealth Uhealth. 2019 Dec 5;7(12):e14473. doi: 10.2196/14473 (PMC6923760; doi:10.2196/14473)
Supplement: Multimedia Appendix 3 [file mhealth_v7i12e14473_app3.pdf]

Multimedia Appendix 3

Table MA3-a. ANOVA on rank and Kruskal-Wallis test results with respect to sleep pattern-related features (Modality 1) among five derived clusters of insomnia sufferers († $P<.1$, * $P<.05$, * $P<.01$, *** $P<.001$).

ANOVA on Rank	Group	Kruskal-Wallis (*P*-value)
sleep_start_time	A-B	.39
F(4,37)=1.74,	A-C	1.00
P=.16	A-D	1.00
	A-E	.39
	B-C	.47
	B-D	.39
	B-E	.19
	C-D	1.00
	C-E	.39
	D-E	.39
sleep_end_time	A-B	1.00
F(4,37)=0.51,	A-C	1.00
P=.73	A-D	1.00
	A-E	1.00
	B-C	1.00
	B-D	1.00
	B-E	1.00
	C-D	1.00
	C-E	1.00
	D-E	1.00
sleep_min	A-B	.35
F(4,37)=2.36†,	A-C	.28
P=.07	A-D	.75
	A-E	.28
	B-C	.28
	B-D	.28
	B-E	.19
	C-D	.47
	C-E	1.00
	D-E	.28
sleep_efficiency	A-B	.752
F(4,37)=9.05***,	A-C	.862
P<.001	**A-D***	**.029**
	**A-E****	**.005**
	B-C	.789
	**B-D****	**.006**
	**B-E****	**.005**
	**C-D***	**.042**
	**C-E***	**.029**
	**D-E***	**.029**

Table MA3-b. ANOVA on rank and Kruskal-Wallis test results with respect to sleep pattern-related features (Modality 2) among five derived clusters of insomnia sufferers († $P<.1$, * $P<.05$, * $P<.01$, *** $P<.001$).

ANOVA on Rank	Group	Kruskal-Wallis (*P*-value)
active_calorie F(4,37)=8.16***, *P*<.001	**A-B***	**.040**
	**A-C***	**.040**
	**A-D****	**.002**
	A-E	.718
	B-C	.248
	**B-D***	**.040**
	B-E	.160
	C-D	.862
	C-E	.106
	**D-E***	**.040**
Walks F(4,37)=6.53***, *P*<.001	A-B	1.00
	**A-C****	**.009**
	**A-D†**	**.074**
	A-E	1.00
	**B-C****	**.009**
	**B-D***	**.018**
	B-E	1.00
	**C-D****	**.009**
	**C-E***	**.032**
	**D-E†**	**.061**
Stairs F(4,37)=3.51*, *P*=.02	A-B	.164
	**A-C†**	**.077**
	A-D	.131
	A-E	.718
	B-C	.189
	B-D	1.00
	B-E	.343
	C-D	.215
	C-E	.215
	D-E	.395

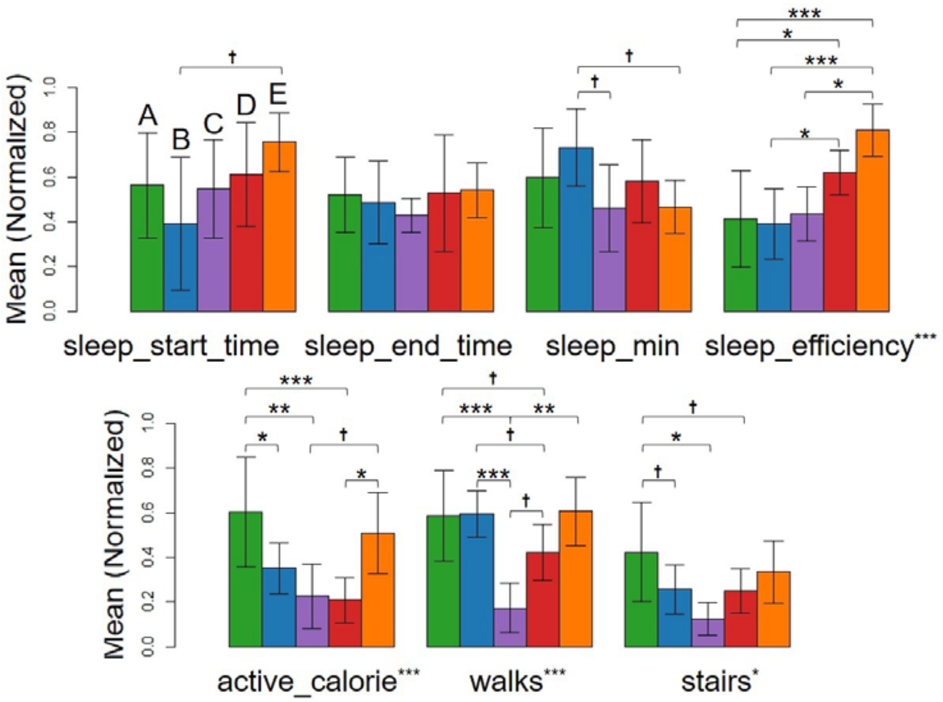

Figure MA1. Bar plots of the major smartband features per cluster († P<.1, * P <.05, ** P <.01, *** P <.001 in ANOVA and post hoc Tukey's honest significant difference (HSD) test results for the derived 5 groups, also find Table MA4-a and MA4-b).

Table MA4-a. ANOVA and post-hoc Tukey's honest significant difference (HSD) test results with respect to sleep pattern-related features (Modality 1) among five derived clusters of insomnia sufferers ($^{\dagger}P<.1$, $^{*}P<.05$, $^{**}P<.01$, $^{***}P<.001$).

ANOVA	Group	Difference	*P*-value
sleep_start_time	A-B	0.17	.55
F(4,37)=1.87,	A-C	0.01	.999
P=.14	A-D	-0.05	.99
	A-E	-0.20	.61
	B-C	-0.16	.84
	B-D	-0.22	.30
	**B-E**	**-0.37**†	**.096**
	C-D	-0.07	.99
	C-E	-0.21	.74
	D-E	-0.14	.82
sleep_end_time	A-B	0.03	.995
F(4,37)=0.25,	A-C	0.09	.93
P=.91	A-D	-0.01	.999
	A-E	-0.02	.999
	B-C	0.06	.99
	B-D	-0.04	.99
	B-E	-0.05	.99
	C-D	-0.10	.92
	C-E	-0.11	.92
	D-E	-0.01	.999
sleep_min	A-B	-0.14	.56
F(4,37)=1.997,	A-C	0.14	.77
P=.12	A-D	0.02	.999
	A-E	0.13	.74
	**B-C**	**0.27**†	**.099**
	B-D	0.15	.45
	**B-E**	**0.27**†	**.099**
	C-D	-0.12	.84
	C-E	-0.01	.999
	D-E	0.12	.82
sleep_efficiency	A-B	0.02	.997
F(4,37)=7.68***,	A-C	-0.02	.999
$P<.001$	**A-D**	**-0.20***	**.04**
	**A-E**	**-0.40*****	**<.001**
	B-C	-0.04	.99
	**B-D**	**-0.23***	**.03**
	**B-E**	**-0.42*****	**<.001**
	C-D	-0.18	.33
	**C-E**	**-0.38***	**.014**
	D-E	-0.19	.21

Table MA4-b. ANOVA and post-hoc Tukey's honest significant difference (HSD) test results with respect to daily activity-related features (Modality 2) among five derived clusters of insomnia sufferers ($^{\dagger}P<.1$, $^{*}P<.05$, $^{**}P<.01$, $^{***}P<.001$).

ANOVA	Group	Difference	*P*-value
active_calorie	**A-B**	**0.25***	**.03**
F(4,37)=8.54***,	**A-C**	**0.38****	**.008**
$P<.001$	**A-D**	**0.39*****	**<.001**
	A-E	0.10	.86
	B-C	0.13	.78
	B-D	0.14	.40
	B-E	-0.16	.55
	C-D	0.02	.999
	**C-E**	**-0.28**†	**.099**
	**D-E**	**-0.30***	**.03**
walks	A-B	-0.01	.999
F(4,37)=7.04***,	**A-C**	**0.41*****	**<.001**
$P<.001$	**A-D**	**0.16**†	**.099**
	A-E	-0.02	.999
	**B-C**	**0.42*****	**<.001**
	**B-D**	**0.17**†	**.099**
	B-E	-0.01	.999
	**C-D**	**-0.25**†	**.08**
	**C-E**	**-0.43****	**.002**
	D-E	-0.18	.22
stairs	**A-B**	**0.17**†	**.099**
F(4,37)=3.54*,	**A-C**	**0.30***	**.02**
P=.02	**A-D**	**0.18**†	**.08**
	A-E	0.09	.83
	B-C	0.13	.65
	B-D	0.01	.999
	B-E	-0.08	.90
	C-D	-0.12	.66
	C-E	-0.21	.31
	D-E	-0.09	.85

< end of document >
